# Understanding systemic land use dynamics in conflict-affected territories: The cases of Cesar and Caquetá, Colombia

**DOI:** 10.1371/journal.pone.0269088

**Published:** 2022-05-31

**Authors:** Martha Lilia Del Río Duque, Tatiana Rodríguez, Ángela Patricia Pérez Lora, Katharina Löhr, Miguel Romero, Augusto Castro-Nunez, Stefan Sieber, Michelle Bonatti

**Affiliations:** 1 Research Area 2 "Land Use and Governance", Leibniz Centre for Agricultural Landscape Research (ZALF), Müncheberg, Brandenburg, Germany; 2 International Institute for Applied Systems Analysis (IIASA), Laxenburg, Austria; 3 Alliance Bioversity–CIAT, Palmira, Valle del Cauca, Colombia; 4 Department of Agricultural Economics, Humboldt Universität zu Berlin, Berlin, Germany; University of Maryland at College Park, UNITED STATES

## Abstract

In the Colombian context, disputes over natural resources, mainly over land, and poor governance are intertwined with armed conflict. Although efforts to address this situation, including the 2016 peace agreement signed between Colombian government and the Revolutionary Armed Forces of Colombia (FARC, by Spanish acronym) are underway, these disputes continue, affecting land use dynamics. Understanding the complexity and trends in land use conflicts, as well as the specific regional characteristics underlying differing land use changes across regions, is critical. This article aims to systematically understand land use dynamics in two contrasting and conflict-affected territories in Colombia, Caquetá and Cesar, thus identifying entry points to address land-use conflicts at the regional level. To address the complexity of each regional case, we apply a methodology based on system thinking to capture the interconnections between socio-economic and environmental system components and their land use dynamics. Results depicted through causal loop diagrams not just show the cascade of environmental, social, and economic failures resulting from land use changes in these two conflict-affected territories but also suggest that land tenure systems innovations and the promotion of sustainable land use interventions at the regional level can reverse the consequences of the land use changes. Thus, future actions addressing land use conflicts must be context-dependent, tackling the root and structural causes.

## Introduction

Land use change is a major driver of global change. Increasing CO2 emissions, changing water cycles, and the loss of biodiversity are examples of the effects of land use change [1–4]. Land use changes and related land use conflicts are closely linked to land tenure systems, especially since the set of property rights associated with the land and the institutions that uphold these rights to guarantee equitable and secure land access are foundational for social stability, economic growth, environmental conservation, and human development [5–7]. Without them, land use conflicts could emerge as a result of competing demands for present and future land uses [8]. In the global south, land use conflicts explode over issues related to social inequalities [8], even triggering armed conflict. This, in turn, could increase or ameliorate the pressure over natural resources and further land use changes [9–11]. For example, countries like Vietnam and Mozambique have experienced massive deforestation during conflict [12]. In contrast, civil war in other countries like Sierra Leone [13] and El Salvador [14] are related to favorable effects of forest cover.

In the Colombian context, disputes over natural resources, mainly over land, and poor governance are intertwined with armed conflict [15–17]. In fact, land distribution inequality is considered to be the root of the armed conflict [18]. This inequality has accelerated land grabbing through coca cultivation and conversion to cattle ranching as a strategy of land accumulation, land speculation, and money laundering by non-legal actors [18–20]. However, the implications of armed conflict show a dichotomous effect on natural resources across regions. For example, the armed conflict generated violent land grabbing, illicit economies, and deforestation in some areas of the country, causing severe environmental damage and boosting unsustainable land uses; simultaneously, the restricted access to some regions due to the presence of armed groups and forced human displacement have allowed biodiversity protection and forest resurgence [10, 16, 21–24].

These processes continue even after the peace agreement. Suarez *et al*. [25], Armenteras *et al*. [26], Clerici *et al*. [10], Zúñiga-Upegui *et al*. [27] find that natural resources extraction, agrarian expansion, and deforestation tended to intensify after the signing of the peace agreement with the Revolutionary Armed Forces of Colombia (FARC, by Spanish acronym) in 2016. Other authors state that forests that were previously inaccessible are now open to land grabbing, in particular with conversion to cattle pastures as a result of land speculation and land accumulation [18, 19]. However, according to Negret *et al*. [23], this tendency depends on regional features such as local governance.

Against this background, the current situation of land use conflicts poses a critical challenge in the Colombian post-agreement context. To understand land use changes in conflict-affected territories in Colombia, a systematic exploration of the complex land use dynamics is required. Few authors map this complexity using causal loop diagrams (CLDs). For example, the study of Rocha *et al*. [28] uses CLDs to understand land use changes in Latin America across different cases. They recognize that CLDs allow for reconciling common regional drivers with context-dependent features. Furthermore, Molina *et al*. [29] develop CLDs to improve the systemic understanding of factors affecting sustainability in the agro-ecosystems of the *páramos* in Colombia. This tool allows them to integrate different elements and feedbacks of these agro-ecosystems, thus broadening discussions about future effects and expected behaviors. Another highly insightful study is that of Arias-Gaviria *et al*. [30]. The scholars analyze deforestation in Colombia using the conceptual framework of socio-ecological systems and CLDs as a tool for mapping interactions between different sub-systems and agents. This study contributes to a better understanding of the complexity of deforestation, a key issue related to land use dynamics in Colombia.

Since the complexity of land use changes is determined by regional patterns [31], it is important to expand its study to regional research that considers context-dependent features. To this end, the paper addresses two main questions: (1) what are the systemic land use dynamics in two strategic and contrasting case studies: Cesar and Caquetá, and (2) how can systemic innovation address land use conflicts?

Our paper contributes to the existing literature by analyzing land use dynamics under a complex system approach that comprehensively maps the interactions and feedbacks related to land use changes. Through a case study comparison and using causal loop diagrams (CLDs) our paper sheds light on the land use dynamics in these two study regions. These CDLs also contribute to a better understanding of the interrelation between three subcomponents of the system: environmental, social, and economic [30, 32–34].

## Case study background

**Fig 1:**
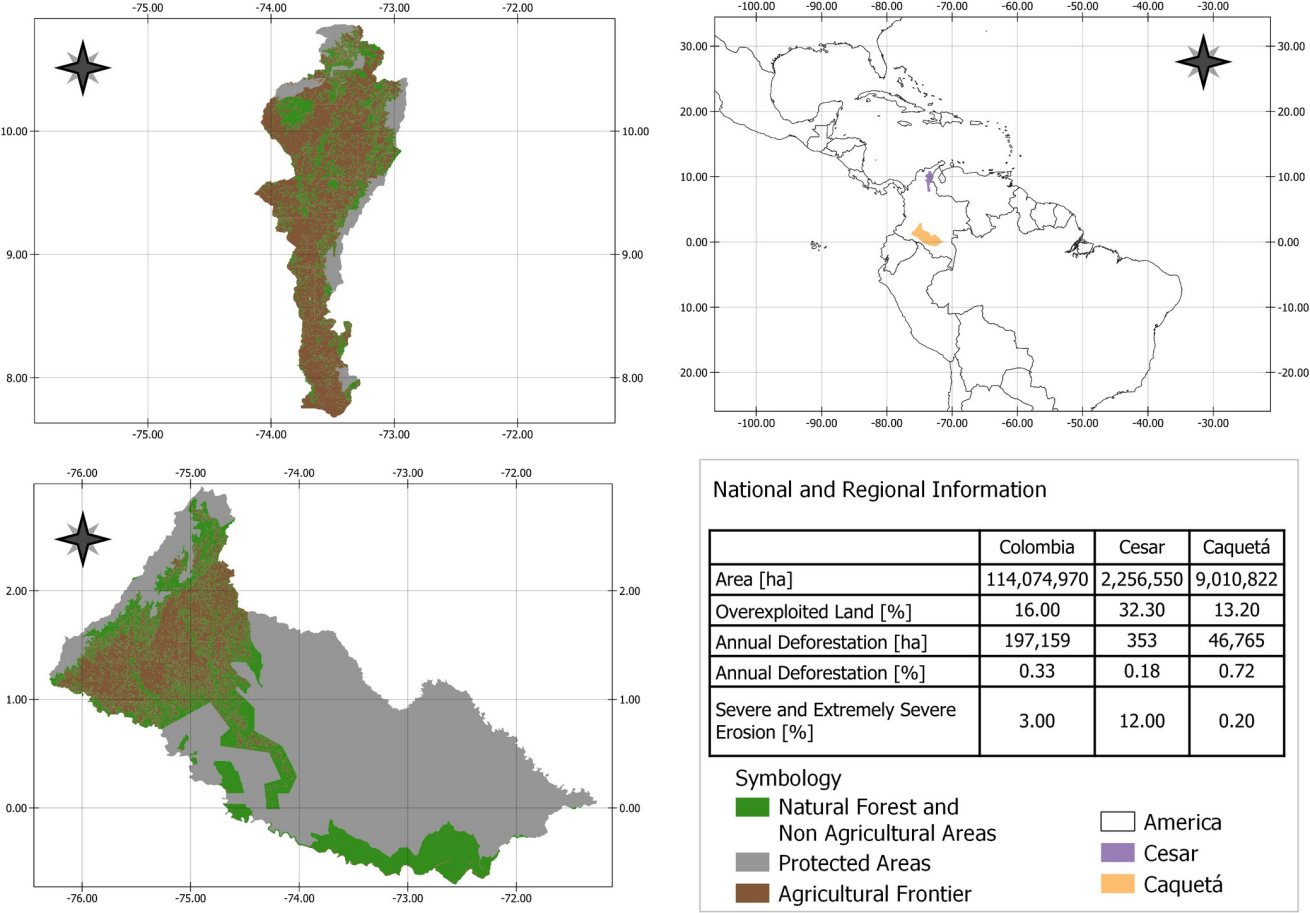
Study regions: Departments of Cesar and Caquetá. Elaborated by authors. America map obtained from GADM (free available at: https://gadm.org/), and the Agricultural frontier of Cesar and Caquetá shape obtained from *Unidad de Planificación Rural Agropecuaria–UPRA* (free available at: https://sipra.upra.gov.co/). The data contained in the table was taken from the following official sources in Colombia: SIPRA, UPRA; IGAC, SMBYC, IDEAM.

Colombia is located on the northwestern end of South America at latitude 4°06’56.42" N and longitude of 72°55’48.49"W. Colombia comprises 32 departments and the Capital District of Bogotá. Departments are subdivided into municipalities. The departments, in turn, can be grouped into six very distinct natural regions: Caribbean, Andean, Pacific, Orinoquía, Amazon, and Insular. We selected two conflict-affected departments of Colombia as case studies: one located in the Amazon region, Caquetá. This region covers 132,218 km^2^, or 11.6% of the country’s surface. The other, Cesar, is located in the Caribbean region. This region covers 403,348 km^2^, or 35.3% of the country´s surface (Fig 1).

Caquetá, located in the southern region, is the third largest department in Colombia with an area equivalent to 7.8% of the national territory, has an important role at national level due to its unique biological corridors and great variety of fauna and flora: it is considered to be one of the most mega-biodiverse departments in the country [35]. As this department is dominated by the Amazonian rainforest, land-based production activities are constrained. In this sense, 63.4% of its area is natural protected area and 20.5% are natural forest and non-agricultural areas [36]. However, historically Caquetá was integrated into the country through successive processes of colonization since the mid-nineteenth century, a process that stimulated the non-vocational use of the land and prioritized extractivism [37]. Further, since its geographical location was suitable, the Revolutionary Armed Forces of Colombia (FARC) established its military activities there, thus making the armed conflict a long-standing central factor in its socio-spatial configuration. Not only was the department the scene of two failed peace negotiations during the governments of Belisario Betancur (1982–1986) and Andrés Pastrana (1999–2002), it also witnessed the largest state attempt to defeat the FARC within the Democratic Security policy of the government of Álvaro Uribe (2002–2010). Neither the State nor the FARC ever completely controlled Caquetá. Rather, State action has lagged behind the colonization process, has failed to order the territory, and has been completely substituted by the FARC in some areas [38]. However, the new peace process changed the dynamics within the region, as we explore in this study.

In contrast, Cesar, located in the northern region, is ranked 21 of 33 according to its area (2% of the national territory) and is 95% rural. Despite its small size, it has four strategic ecosystems for conservation: Sierra Nevada de Santa Marta, Serranía del Perijá, the valley of the Cesar River and the valley of the Magdalena River. Although Colombia has lost around 90% of Tropical Dry Forest (TDF)–only 720,000 of 8.8 million hectares estimated remain–Cesar region has the largest volume of TDF remnants in the country, at 72,401 ha, or 21.8% [39]. According to Instituto Geográfico Agustín Codazzi [40], both productivity and conservation could be combined in the department because of its 2.2 million hectares: 48% is suitable for different types of production and 51.2% should be protected due to its environmental importance. Palm oil crops, coal mining, cocoa, and livestock are the main economic activities in the region. Coal mining is particularly causing environmentaland social damages. Nearly all of the intact forest has been destroyed near the mines, the underground aquifers have been permanently damaged, rivers have been diverted away from communities, and there are dangerous levels of air and water pollution [41, 42]. As for the conflict, there were several violent actors in the department and, in contrast to Caquetá, paramilitary groups played a more significant role in Cesar. For example, between 1997 and 2006, the Drummond Company, a mining company, supported with money the leaders of the United Self-Defense Forces of Colombia (*Autodefensas Unidas de Colombia*, or AUC, in Spanish), who were located in the coal mining areas in the north of the department of Cesar. In addition, the Northern Block of the AUC, under the leadership of Jorge 40, a member of the elite Cesar department, sought to capture elected offices in some Colombian Atlantic coast departments, including Cesar [43].

Although land use changes in both departments have developed differently and differing land suitability, they have some similar characteristics that allow for the comparison of both departments. First, in both regions, resource depletion is associated with land accumulation and the expansion of the agricultural frontier.

Second, both regions were highly affected by armed conflict and, for this reason, some territories within these two regions are included in the Territorially Focused Development Programs (PDETs, by Spanish acronym). These programs are an instrument that aims to stabilize and transform the territories most affected by violence, forced displacement, poverty, illicit economies, and institutional fragility in Colombia [44].

Thirdly, these departments present high rural land concentration. According to UPRA [45], Cesar and Caquetá are two of the five departments with the greatest rural properties and tend to have the highest variability and the largest median farm sizes. That is, properties in these departments are less similar in size. Fourth, most of Caquetá and Cesar have land tenure issues, with 58.7% of rural land in Caquetá being technically and legally informal or imperfect; in Cesar it is 47.43%; this makes it difficult to access institutional support [46]. Fifth, land use change in both cases resulted in the establishment of extensive pastures for cattle ranching. Sixth, in both regions there is a growing interest in developing other crops, including cocoa. Seventh, and finally, inequality in access to rural land is high for both departments. Thus, the Gini index for 2014 in Cesar was 0.7 and for Caquetá it was 0.6 [45]. This index is one of the most widely used indicators reflecting the level of inequality in land distribution. The indicator shows how the land area is distributed among its owners. Values close to one indicate high inequality in property distribution. Egalitarian distributions would show a Gini index close to zero, where, for example, 10% of the owners have 10% of the area. The estimation for Colombia as a whole is even more drastic than in the two case studies, at 0.8789, which represents very high inequality in the property land distribution [45].

## Materials and methods

### System dynamics and Causal Loops Diagrams (CLDs)

System dynamics, widely believed to be critical for handling the complexity facing the world, comprises a set of conceptual tools that enable understanding the structure and dynamics of complex systems [47]. In other words, system dynamics allows for taking a problem apart and reassembling it to understand its components and causal relationships [48, 49]. CLDs are a useful tool within this approach, helping to provide a broad range of insights and implications [50] that can be used as the basis for developing actions and implementing policy [51]. CLDs link key elements, highlighting the causal relationships between them through loops. By stringing together several loops in these diagrams, it is possible to visualize and explain a logical interconnected chain of facts that creates a story about a complex situation and that even shows systemic failures. In this context, a systemic failure refers to a failure in one part of the system or parts of the system that propagates through the whole system and impacts the entire interconnected chain of social, economic, and environmental events [52].

### Methodological approach

**Fig 2:**
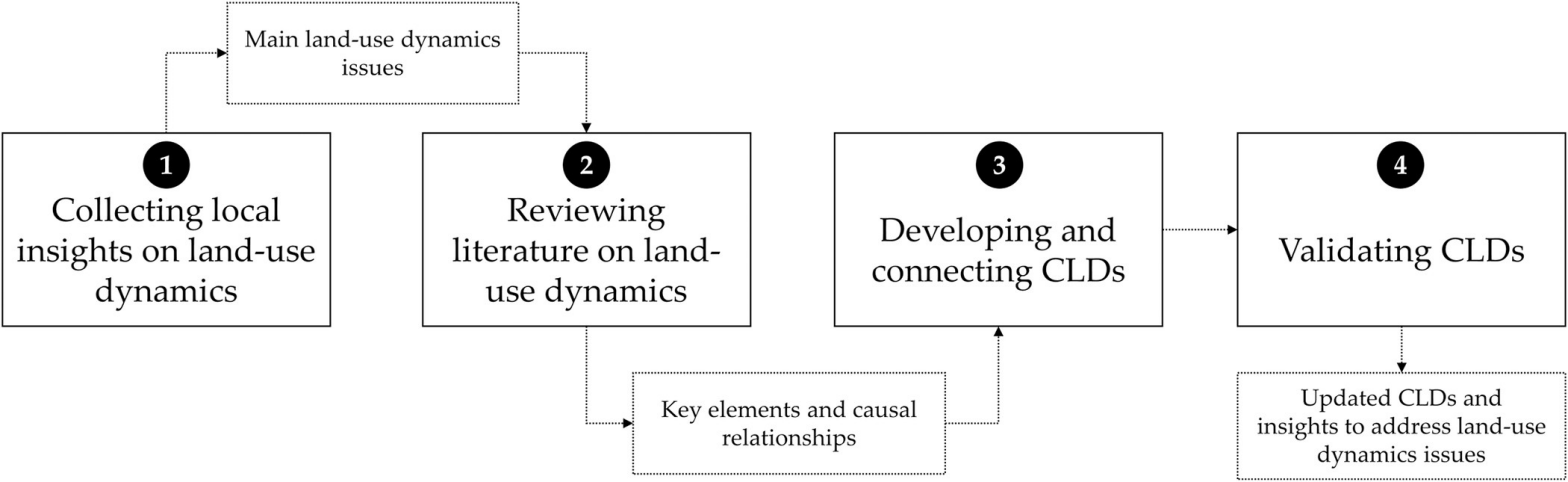
Methodology.

Here, we describe the four steps taken to build and validate the regional CLDs using primary and secondary sources in order to understand their land-use dynamics (Fig 2). All participants involved in the study were informed of its objectives and they gave their verbal informed consent to participate in the interviews or workshops. The study was approved by the Institutional Review Board (IRB) of the Alliance Bioversity-CIAT, which complies with international ethical standards.

The research was principally conducted by a group of Colombian experts on sustainable land uses. As a first step, to advance the existing knowledge within the group, we collected other experts’ knowledge insights on several issues related to land use dynamics of the study regions. For this, we conducted four initial qualitative in-depth semi-structured interviews in December 2019. To consider diversity of knowledge and experience in the research topics, the criteria to select the experts were: (1) experience on activities related to land use dynamics in the areas analyzed; and (2) knowledge about land tenure systems. Therefore, we selected experienced experts on development cooperation projects, representatives of NGO’s and community-based organizations. The first interviewee was a practitioner with experience in implementing land formalization and restitution projects around Colombia. The second interviewee was a representative of a local NGO in Caquetá with knowledge and experience on the patterns of land-use change and deforestation in the Amazon region. The third interviewee was a farmer and social leader, who has been involved in sustainable rural development and social movements against land grabbing in Cesar. The semi-structured interviews included three guiding questions: (1) what is your perception about land informality and land restitution processes in each department?; (2) what are the current land use conflicts that are affecting each department?; and (3) do you know formal institutions or mechanisms to solve land use conflicts in each department? Each interview lasted between 60 and 90 minutes. All interviews were transcribed and data analyzed using qualitative content analysis, a method to identify manifest and latent structures in texts and other qualitative material [53, 54].

Secondly, based on the expert insights we conducted a literature review to identify key elements and causal relationships on the main land-use dynamics issues in each department. Like Schlindwein and Ison [52], we retrieved information from two sources: peer-reviewed articles and reports from different national and international institutions. Regarding the scientific literature search, it was conducted through Scopus using search equations with keywords for each study area. For Caquetá, we used the following equation: TITLE-ABS-KEY ("land-use change" OR "land-use conflicts" OR deforestation) AND TITLE-ABS-KEY ("Caqueta" OR "Amazon") AND TITLE-ABS-KEY (Colombia). For Cesar, we employed the combination of the following words: TITLE-ABS-KEY ("land-use change" OR "land-use conflicts" OR "degradation") AND TITLE-ABS-KEY ("Cesar" OR "dry forest") AND TITLE-ABS-KEY (Colombia). The results contained 123 studies from those two sources aforementioned and finally, 76 documents were both relevant and fully reviewed. Their content were analyzed and categorized into three main codes related to the three subcomponents of the system: environmental, social, or economic.

Thirdly, we connected the key elements and causal relationships by developing the first version of CDLs using the steps of Cavana and Mares [55]: (1) identify and create key elements that can be measured and monitored; (2) establish links between the key elements in the CLDs; (3) indicate the polarity of each link using no sign if the effect goes in the same direction or “-” if it goes in the opposite direction; and (4) identify and label the loops as reinforcing (R) when it is an amplifying or enhancing feedback loop or balancing (B) when it is a stabilizing, goal seeking, or regulating feedback loop [56].

Fourthly, we presented a draft version of CLDs in two virtual validation workshops with experts in land use interventions. The first one took place in May 2020 and lasted 120 minutes. It was attended by 13 researchers who were working on interdisciplinary projects related to sustainable land uses in the study areas. The second workshop, occurring in April 2021, lasted 90 minutes and comprised 16 researchers with the same expertise as the first workshop group, participated. During the workshops, we presented the CLDs and encouraged the discussion using the following guiding questions: (1) were the most important elements represented in the CLDs or do we need to introduce or delete elements?; (2) do we have the most important interactions, or do we need to add or remove any?; (3) do we have the most important loops, or do we need to add or remove any?; and (4) does the CLDs offer a counterintuitive or intuitive insight of processes that really occur in the study area? The interventions of participants were transcribed and data analyzed to refine the draft version of CLDs by incorporating or removing key elements, links between key elements or loops. They also generated insights about how to address land-use conflicts through systemic innovation and promotion of sustainable land use management interventions at the regional level.

After these two rounds of validation we obtained the final CLDs for each department, depicting the consensus points around land use dynamics.

## Results

The data generated through the interviews and workshops allowed us to create a list of key entry points concerning their land use dynamics and conflicts that were analyzed in light of the literature, represented in causal loops diagrams and validated by experts in land use interventions. These entry points are summarized in Table 1.

**Table 1:** Key entry points on land use dynamics in Caquetá and Cesar.

Component	Caquetá	Cesar
Social/Institutional	Lack of governance in the territory facilitates land grabbing	Lack of land tenure formalization
	Weak institutional capabilities and low infrastructure to promote value chains	Land restitution is a complex problem because of the conflict was used to accumulate land illegally. These lands were used for cattle ranching, oil palm and also for coal mining.
	Land tenure system issues lead to unsustainable land uses and deforestation.	There are conflicts due to extensive cattle ranching
	Extensive cattle ranching was even promoted by government in previous decades.	Lack of natural resources use planning
Economic	Land grabbing for extensive cattle ranching or illicit crop cultivation	Lack of access to credit hinders sustainable land planning
	Markets that promote unsustainable land uses, deforestation and expansion of the agricultural frontier	
	Lack of access to credit for promoting sustainable livestock systems	
	Land grabbing is one of the ways of money laundering.	
Environmental	Growing deforestation and low institutional capacity to curb it	There are conflicts in the communities for the availability and use of water resource. Palm oil cultivation is a driver of conflict due to the large-scale use of this resource. Coal mining has affected water availability.
		Land degradation and lack of access to water increase the risk of agriculture under climate change scenarios, lead to lower productivity and promote the expansion of the agricultural frontier.
		High land degradation by erosion due to inadequate land uses

The overview shows land use conflicts in Caquetá are heterogeneous and depend on if they are in the foothills or not. There are different factors that influence these conflicts, among them: armed conflict, unsuitable land uses, competition between land uses or an overlap between these different factors. On the other hand, land grabbing is one of the ways to launder money and therefore the issue of illicit crops is related to deforestation, conflict, cattle ranching and unsustainable land uses. Thus, the promotion of sustainable land uses in the department is related to the legality of land tenure. In the case of Cesar, poor land use planning and public policy promoted crops such as cotton and oil palm in the department. These types of unsustainable uses degraded the land and resulting in an excess of depleted land dedicated to extractive and extensive cattle ranching. On the other hand, equity in access to water is a fundamental issue in this department. It is notable that the conflict in Cesar is closely related to the issue of land accumulation and concentration.

### Systemic implications of land use conflicts in Caquetá

**Fig 3:**
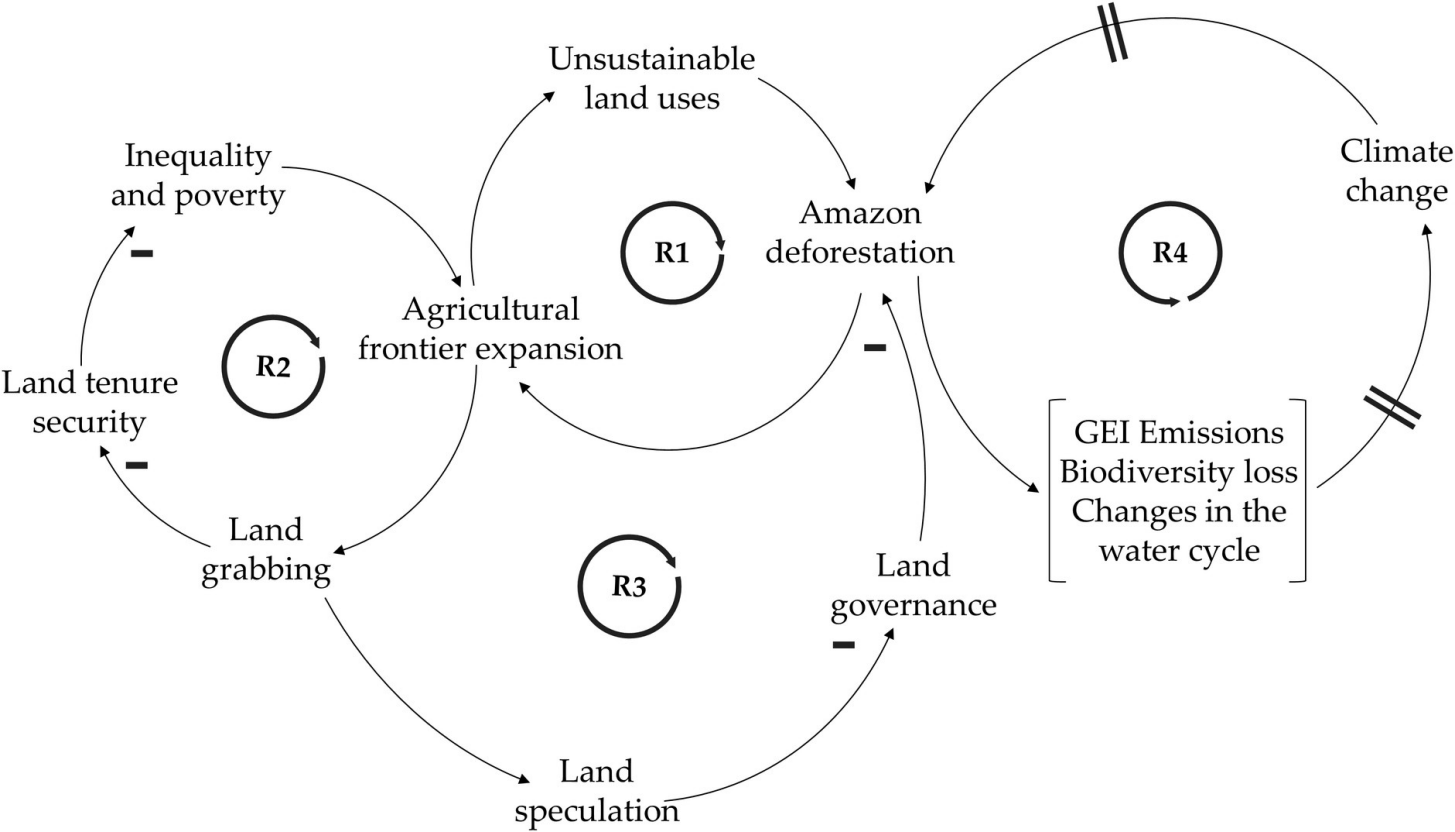
Causal loop diagram of land use conflicts in Caquetá, Colombia. Key elements are joined by arrows indicating where there is a causal relationship between them. A (-) sign adjacent to an arrow indicates that the cause has a negative effect; if the cause has a positive effect there is no sign. The encircled R in each loop center means that the loop is a reinforcing loop (positive feedback). The equal sign located on the arrows symbolizes a delay in the system.

Fig 3 depicts land use conflicts in Caquetá through four reinforcing loops related to (R1) agricultural land-use conflicts and deforestation, (R2-R3) their implications on the land tenure system; and (R4) climate change.

Caquetá has historically generated conditions fostering legal and illegal land colonization. This process, which has been poorly planned and uncontrolled, led to the deforestation of the Amazon rainforest [57, 58]. Nowadays, Caquetá is one of the most deforested departments in Colombia. Overall, Colombia lost 197.159 ha of forest in 2018, and for Caquetá alone that value was 46,000 ha. This department ranked first in deforestation, followed by Meta (44,712 ha), Guaviare (34,527 ha) and Putumayo (13,903 ha), all of them are located in the same geographical region [59], among other examples. In most cases, this deforestation is caused by external settlers who expand the agricultural frontier by clearing the tropical forest, which in turn triggers the establishment of unsustainable land uses, such as illicit crops, mining, and extensive cattle ranching [23, 60–62]. As the land is not suitable for these uses, its poor productive capacity is depleted and deforestation continues [63], as depicted in the reinforcing loop R1. The process of agricultural frontier expansion is an essential element stimulating the land grabbing schemes in the department where settlers invade the land, mainly for converting forest to pastures for cattle raising to get a formal title, and which is subsequently exploited by large landowners who buy the titles [20]. This process exacerbates the well-known problem of land tenure insecurity and concentration that, in turn, increases inequality and poverty since smallholders lack capital and credit to buy already cleared lands. These land-related problems create a perverse incentive for expanding the agricultural frontier by clearing more forests [26], as reinforcing loop R2 shows. Reinforcing loop R3 illustrates the implications of land grabbing, which in Colombia is considered to be a way for large investors and armed actors to transform illegal assets into legitimate capital [64]. This process generates land speculation that diminishes land governance due to power asymmetries and land inequality, thus the lack of governance over natural resources in the region facilitates increasing deforestation. The reinforcing loop R4 reveals how the ongoing Amazon deforestation creates a set of negative environmental effects, including the increase of greenhouse emissions [65], biodiversity losses, and water cycle changes, that subsequently exacerbates climate change. These changes could intensify the deforestation in the long term, since climate change, droughts and rising temperatures may contribute to the death of vulnerable trees in the forest, thus creating a new cycle causing further deforestation [66].

### Systemic implications of land use conflicts in Cesar

**Fig 4:**
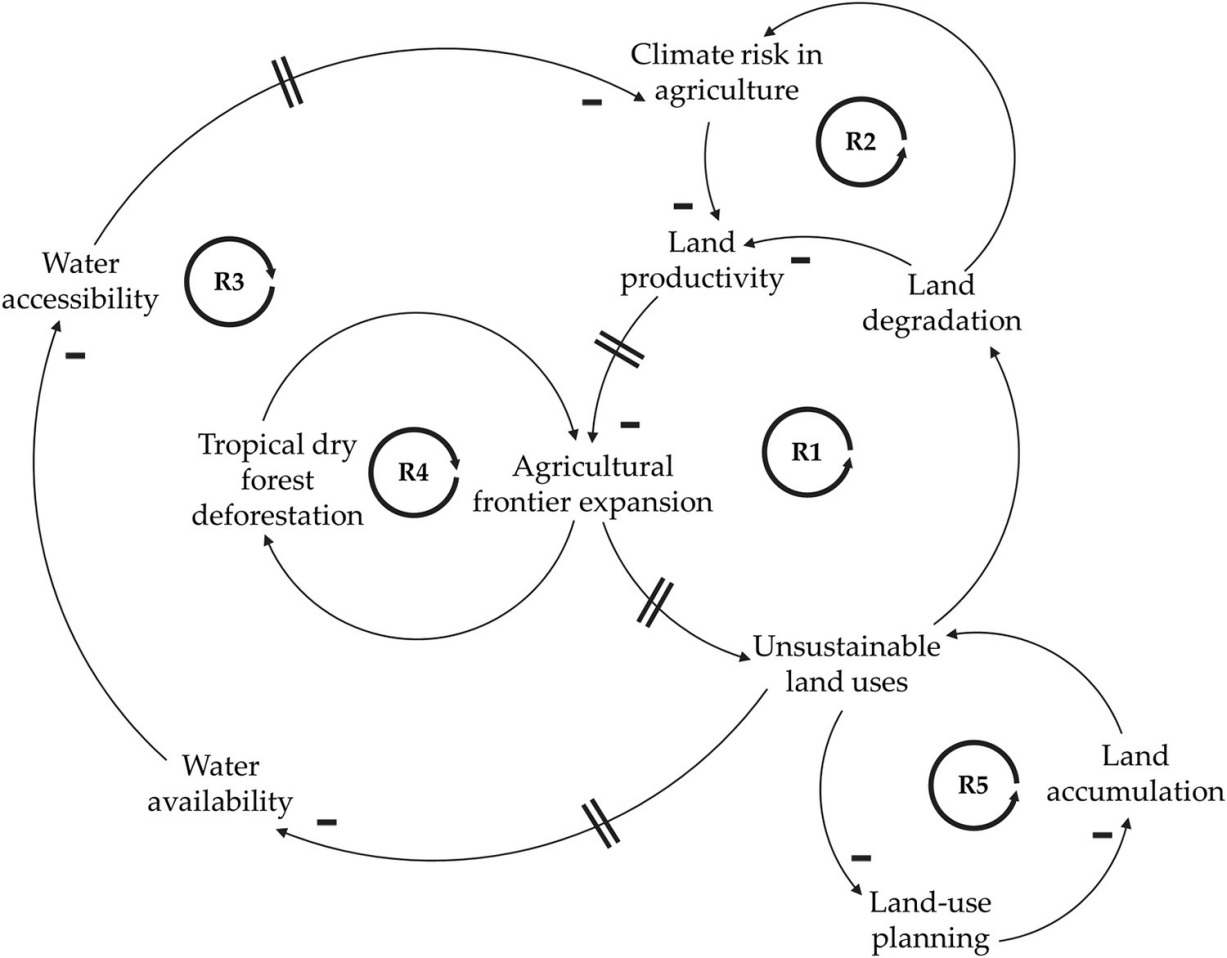
Causal loop diagram of land use conflicts in Cesar, Colombia. Key elements are joined by arrows indicating where there is a causal relationship between them. A (-) sign adjacent to an arrow indicates that the cause has a negative effect; if the cause has a positive effect there is no sign. The encircled R in each loop center means that the loop is a reinforcing loop (positive feedback). The equal sign located on the arrows symbolizes a delay in the system.

Fig 4 shows the systemic structure of land use conflicts by means of different reinforcing loops regarding unsustainable land uses and land degradation (R1), climate risk in agriculture (R2), land productivity and water management (R3), forest conservation, agricultural frontier and resource depletion (R4) and land tenure system (R5).

The reinforcing loop R1 exhibits how land productivity is directly affected by land degradation, which is evidenced by the increasing soil erosion in the region. The probability of keeping productive systems in areas with desertification and overexploitation processes is very low [67]. In 2021, Cesar has some of the most degraded soils in Colombia, with 81.9% of erosion. This degradation by erosion specifically affects agricultural yields, with 60.4% of all agricultural sites eroded to some extent [68]. Due to the non-vocational use of land, productivity constraints are increasingly evident because people incorrectly believe they can adapt soils to agricultural uses instead of the other way around [68]. With regard to the effects of erosion on climate change, as illustrated in the reinforcing loop R2, FAO [69] points out that the loss of soil organic carbon not only negatively affects soil health and food production, but also exacerbates climate change and the risk of damage in the agricultural sector. As Selvaraju [70] remarks, agriculture is deeply interconnected with weather and climate, making this factor dominate in the overall variability of food production and a continuing source of disruption to ecosystem services and facilitator of land use conflicts.

Concerning the reinforcing loop R3, unsustainable land uses in the region has also affected water availability. Inadequate patterns for access, occupation, and use of natural resources, as well as poor technological practices, are threatening their valuable natural endowments [42]. Currently, the water deficit and the low water quality in mining zones represent a critical future scenario for the preservation of fauna and flora, for food security and for the development of agricultural value chains, such as palm oil, coffee, livestock, and cocoa, among others [42, 71]. Under climate change scenarios, with more severe droughts, water accessibility problems increase risk in the agricultural sector. Another cascade of impacts are found when looking at the effects of decreasing agricultural productivity on the conservation of tropical dry forest remnants, as depicted in the reinforcing loop R4. In Cesar, strategic ecosystems, such as tropical dry forest and swamplands, lack a management strategy that takes into account their conservation, ecologic dynamics and ecosystem services [67]. Therefore, they are heavily degraded, as a result of the diverse pressures that human beings have exerted on it, including deforestation with the purpose of economic exploitation, coal mining, agricultural expansion, and cattle ranching [39, 41, 42, 72, 73]. Finally, the cascade of failures in the reinforcing loop R5 highlights the lack of appropriate land use planning that takes into account the land suitability. This precarious land use planning, including land tenure, land access, and land distribution, alongside the current land restitution programs, has also led to land accumulation with unsustainable uses and, consequently, with more soil degradation by erosion [68]. This last social reinforcing loop related to the land tenure systems is particularly important in Cesar since the inequality in land access helped to consolidate extractive activities and palm oil monocultures, which in turn represented a driver for disputes between armed actors for territorial control and resource extraction [74–76]. Currently, the land tenure issues as land restitution process to displaced victims of the conflict show a slow progress in Cesar. For example, the department has the highest level of land dispossession and has the fifth largest number of requests for land restitution outstanding at the national level, thus perpetuating the insecure property rights situation [76–78].

## Discussion

We map and compare the complexity of the problems related to land-use dynamics in two conflict-affected territories using CLDs. Neither diagram represents a screenshot of a current situation, rather they show how a series of factors that are separated by location and/or time might interact to create systemic failures. By using this tool, we are able to compare the two regions, identifying not just context-dependent characteristics but also systemic common drivers and trends of land-use changes.

Land use changes in conflict-affected territories in Colombia involve complex system where everything is connected to everything else. Causal loops diagrams allowed us to conceptualize and construct our circular connections, mutual causality and the feedbacks in our problem. They are grounded in the theory of nonlinear dynamics and their feedbacks suggest that everyone shares responsibility for problems generated by a system. Thus no “one” factor is solely responsible for changes in the land use system [79–82].

A common driver of land-use change has to do with land access and distribution problems, which translate into a high land concentration in both regions. However, it is the result of dissimilar pathways that have been followed in each region. Land grabbing by clearing forests is how land is accumulated in Caquetá for speculative purposes. Unfortunately, this process was exacerbated following implementation of the peace agreement due to the power vacuum resulting from the demobilization of the guerrillas [23, 57, 83, 84]. Land dispossession caused by forced displacement is the way to accumulate lands in Cesar, which are then converted into economic exploitation areas. Unfortunately, the process to bring the lands back to their previously dispossessed owners, which is known in Colombia as land restitution, is slow and complex since land restitution programs have had limited capacities to distinguish between those who used violence to accumulate land and those who did not [85, 86]. Both land grabbing in Caquetá and land dispossession in Cesar have brought about unsustainable land-uses that are depleting natural resources (e.g., forest, water, soil). In this sense, the presence of land-use conflicts (e.g., insecure or unclear property rights) or soil degradation represents a critical challenge to assure investments in agriculture and sustainable livelihoods in conflict-affected regions, as also mentioned by Counter [86] and Suarez et al. [25].

With respect to the environmental perspective, we observe a common trend in both CLDs: the establishment of unsustainable land uses is reinforcing climate change by affecting the capacity of the ecosystems in these regions to capture and store carbon, by increasing greenhouse emissions and biodiversity losses, as well as by affecting the water cycles. Both CLDs show how these biophysical changes represent a limitation to develop sustainable productive strategies or ecosystem restoration actions. This set of related situations seem to lead to a systemic failure [52] that affects the whole system in social, productive, and environmental terms.

CLDs are a powerful tool that, beyond describing the dynamics of a complex system, enables envisioning new ways to address the problems arising from the described system dynamics [87]. The formulation of such solutions relies on the spotting of the element(s) that are modifiable and can generate a balancing effect in the loops. Which, in our case studies, translates into the development of actions to balance the unsustainable patterns and conflicts of land use. These actions should simultaneously integrate strategies addressing the common systemic drivers in both departments and solutions accounting for the particular regional land use dynamics [63].

Regarding the systemic drivers of land-use change that are common in both regions, a first set of actions should aim to improve the land tenure system, since inappropriate activities, such as deforestation, extensive cattle ranching, illicit crops, and violent land grabbing, are consequences of contradictory land management legislation and plans that do not have enough incentives to promote sustainable land uses, as Clerici *et al*. [10], Furumo & Lambin [78] and Grajales [64] state.

This set of actions is severely limited by different variables or issues, including armed conflict, informal land tenure, land restitution claims, and land grabbing. It is also worth mentioning that these issues prevail or change across the different departments. For example, Caquetá and Cesar are among the ten departments with the most municipalities in the ranking of most affected zones of the Armed Conflict (ZOMAC) [88]. Additionally, there are also high rates of informal land tenure in Caquetá (59%) and Cesar (47%) [46]. In terms of restitution claims, the problem is most critical in Cesar compared with Caquetá, since 6.2% of the claims at the national level were made in Cesar, while in Caquetá these claims were equivalent to 3.2% [77]. Finally, land grabbing particularly affects the Caquetá, Guaviare, and Meta departments, all located in Amazon region. They have been deeply affected by soaring deforestation sparked by land grabbing [89].

Without addressing land grabbing and weakness of land tenure systems, land use conflicts will continue, as Armenteras *et al*. [26] and Wiig & García-Reyes [76] note. To tackle these current issues, scholars like Elhawary [90], Ramirez [91], and Robinson *et al*. [7] suggest different strategies. Strategies include strengthening and supporting relevant government institutions for protecting land abandoned by forced displacement; supporting the State’s constitutional control bodies to ensure compliance of land protection and restitution; supporting communities in the direct protection of their property rights; as well as assisting internally displaced persons to understand and claim their land rights; effective land reform processes; titling systems that promote tenure security; agrarian jurisdiction courts to resolve disputes; as well as improving, monitoring, and evaluating tenure governance systems; among other strategies. These strategies can also serve as an effective strategic tool for supporting the rural poor, for improving livelihoods of farmers, for curbing speculation in land markets, and for preventing deforestation through market-based conservation mechanisms [7, 91–93].

A second set of actions should focus on systemic innovation and promotion of sustainable land use management interventions at the regional level. As land use dynamics, land degradation, climate change, and rural poverty are linked, these kinds of interventions could stop the cascade of systemic failures caused by land-use conflicts. Sustainable land use management strategies are a set of systemic interventions that address the causal relation between land productivity, land planning, land degradation, deforestation, restoration of natural areas, unsuitable land uses, and armed conflict [57, 76, 94]. However, consistently with other studies (e.g., Wiig & García-Reyes [76]; Furumo & Lambin [78]; Krause [95]), we find that these interventions need to be linked to the first set of actions since structural issues as inequalities in land ownership, the lack of a full cadaster system, and lack of legal land rights must be overcome to produce long-term change, further investments in agriculture and curb deforestation.

**Fig 5:**
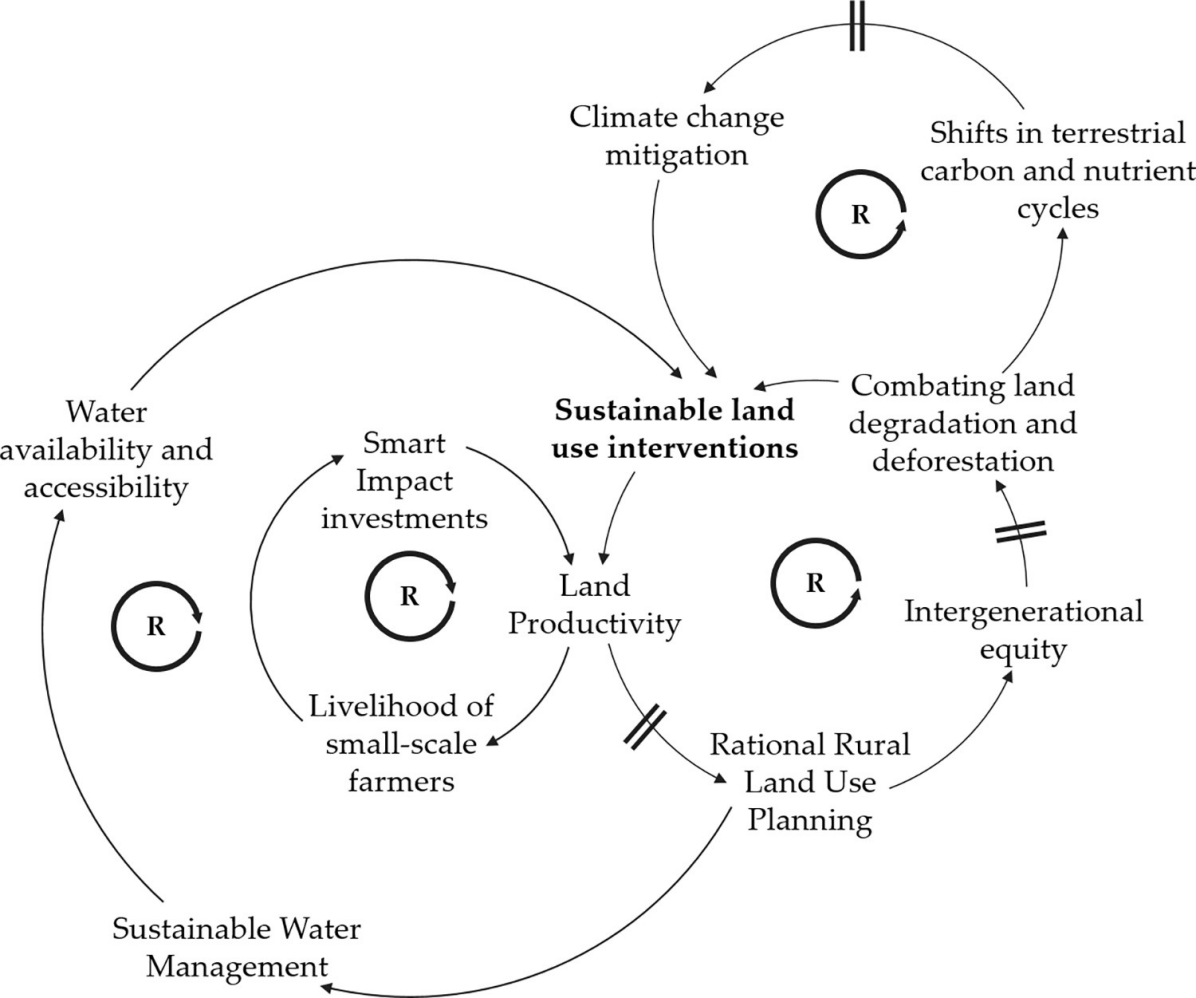
Key factors for effective design and implementation of sustainable land use systems to reduce deforestation and enhance peacebuilding in Colombia. Based on the policy brief elaborated by the same authors: Bonatti *et al*. [96].

The effect of such interventions are illustrated in Fig 5. The CLD shows how the transformative land management practices could avoid systemic failures, by reversing the unsustainable land use patterns of Caquetá and Cesar that are depicted in Figs 3 and 4. Furthermore, through the implementation of these interventions, the soil could be rendered as a sink rather than a source for atmospheric CO2, which is important for decreasing the risk of damage in the agricultural sector.

To carry out these productive interventions, it is necessary to prioritize areas where cost-effective restoration interventions can be implemented. Several studies point out that agroforestry systems are another alternative that restores the land while promoting the sustainable intensification of agricultural production, conservation of native ecosystems, ecological rehabilitation at a landscape scale and carbon sequestration in tropical regions [60, 73, 97–100]. However, these sustainable land use strategies must be integrated into sustainable value chains by establishing policies, institutions, infrastructure, and incentives that facilitate not only the integration of several stakeholders, but also to generate a shared vision towards a sustainable land use and forest restoration [101]. In this regard, for suitable intervention design, the most important lesson to be drawn from complex systems is the need to design and implement interventions that take into account the interconnectedness of systems, as previously observed by Schlindwein & Ison [52].

Finally, the limitations of the study should be considered when observing its results. Although our CLDs aim to systematically understand the dynamics of two different regions, they are limited by local actors’ insights and variables reported in secondary information. Therefore, they could be incomplete with respect to systems dynamics, as Sterman [47] states.

## Conclusions

This study provides a holistic picture of the land-use dynamics in two different conflict-affected regions of Colombia: Caquetá and Cesar. Through the development and the analysis of both regional CLDs, we find shared drivers and particular interactions that triggered unsustainable land uses. Additionally, we identify cross-cutting key elements in the study regions and specific actions that should be considered to comprehensively address disputes over land and their implications.

Land tenure issues are cross-cutting elements that encourage conflicts and unsustainable land use practices in both regions. However, they follow dissimilar paths and have different land-use implications. In Caquetá, land grabbing and speculation comes at the expense of the forest, while in Cesar violent land dispossession facilitated the establishment of extractive activities, palm oil monocultures, and extensive cattle ranching; these trigging a poor performance in the land restitution program. Despite differences in the regional paths, both cases show high land concentration and inequality that, along with a lack of governance, continue to exacerbate unsustainable land uses.

Actions to address these unsustainable patterns of land use change should take into account the interconnectedness of the different system components in both departments. In this regard, addressing land-related issues should be a central pillar for the post-conflict process at the national level by guaranteeing an appropriate rural land tenure system. In addition, local authorities in both regions must promote sustainable land use alternatives to restore degraded lands, reduce deforestation, and improve local livelihoods.

## Supporting information

S1 File (PDF)
