# Supplementary material for: Understanding systemic land use dynamics in conflict-affected territories: The cases of Cesar and Caquetá, Colombia
Source: PLoS One. 2022 May 31;17(5):e0269088. doi: 10.1371/journal.pone.0269088 (PMC9154098; doi:10.1371/journal.pone.0269088)
Supplement: S1 File — (PDF) [file pone.0269088.s001.pdf]

Recolección de información para el análisis de las dinámicas en los usos del suelo en los departamentos de Cesar y Caquetá

La entrevista y/o discusión grupal a continuación hace parte del proyecto de investigación SLUS “Implementando Sistemas de Agricultura y Ganadería sostenibles para la Conservación de Bosques, la Mitigación del Cambio Climático y la Construcción de Paz” el cual es liderado por el Centro Internacional de Agricultura Tropical (CIAT) y co-implementado por el Centro Leibniz para la Investigación del Paisaje Agrícola (ZALF) y CIPAV/InstitutoThünen.

En el marco de este proyecto, Martha Del Río y Tatiana Rodriguez, estudiantes doctorales de la Universidad Humboldt de Berlín y del Instituto ZALF en Alemania, están realizando su investigación con el objetivo de entender sistemáticamente las dinámicas del uso de la tierra en dos territorios contrastantes y afectados por el conflicto en Colombia: Caquetá y Cesar, identificando así los puntos principales para abordar los conflictos de uso de la tierra a nivel regional.

Preguntas orientadoras de las entrevistas semiestructuradas

(1) ¿Cuál es su percepción acerca de la informalidad de la tierra y de los procesos de restitución de tierras en Caquetá/Cesar?

(2) ¿Cuáles son los conflictos actuales de uso de la tierra que afectan a Caquetá/Cesar?

(3) ¿Conoce las instituciones o mecanismos formales para resolver los conflictos de uso de la tierra en Caquetá/Cesar?

Preguntas orientadoras de los talleres en línea

(1) ¿Estaban representados los elementos más importantes en los CLDs o necesitamos introducir o eliminar elementos?

(2) ¿Tenemos las interacciones más importantes, o necesitamos añadir o eliminar alguna?

(3) ¿Tenemos los bucles más importantes, o necesitamos añadir o eliminar alguno?

(4) ¿Ofrecen los CLDs una visión contra-intuitiva o intuitiva de los procesos que realmente ocurren en Caquetá/Cesar?

Data collection for the analysis of land use dynamics in the departments of Cesar and Caquetá

The interview and/or group discussion is part of the SLUS research project "Implementing sustainable agricultural and livestock systems for simultaneous targeting of forest conservation for climate change mitigation (REDD+) and peace-building in Colombia", which is led by Alliance of Bioversity International and the International Center for Tropical Agriculture (CIAT) and co-implemented by the Leibniz Center for Agricultural Landscape Research (ZALF) and CIPAV/Thünen Institute.

In the framework of this project, Martha Del Río and Tatiana Rodriguez, PhD candidates at Humboldt University of Berlin and ZALF in Germany, are conducting their research with the objective of systematically understanding land use dynamics in two contrasting and conflict-affected territories in Colombia, Caquetá and Cesar, thus identifying entry points to address land-use conflicts at the regional level.

Guiding questions of semi-structured interviews

(1) What is your perception about land informality and land restitution processes in Caquetá/Cesar?
(2) What are the current land use conflicts that are affecting Caquetá/Cesar?
(3) Do you know formal institutions or mechanisms to solve land use conflicts in Caquetá/Cesar?

Guiding questions of online workshops

(1) Were the most important elements represented in the CLDs or do we need to introduce or delete elements?
(2) Do we have the most important interactions, or do we need to add or remove any?
(3) Do we have the most important loops, or do we need to add or remove any?
(4) Do the CLDs offer a counterintuitive or intuitive insight of processes that really occur in Caquetá/Cesar?
